# Assessment of Novel Proteins Triggering Celiac Disease via Docking-Based Approach

**DOI:** 10.3390/molecules29010138

**Published:** 2023-12-26

**Authors:** Mariyana Atanasova, Ivan Dimitrov, Antonio Fernandez, Javier Moreno, Frits Koning, Irini Doytchinova

**Affiliations:** 1 Faculty of Pharmacy, Medical University of Sofia, 1000 Sofia, Bulgaria; idimitrov@pharmfac.mu-sofia.bg (I.D.); idoytchinova@pharmfac.mu-sofia.bg (I.D.); 2 European Food Safety Authority, 43126 Parma, Italy; antonio.fernandezdumont@efsa.europa.eu; 3 Instituto de Investigación en Ciencias de la Alimentación (CIAL), Consejo Superior de Investigaciones Cientificas-Universidad Autonoma de Madrid (CSIC-UAM), Campus of Interntional Excellence—CEI (UAM+CSIC), Nicolás Cabrera, 9, 28049 Madrid, Spain; javier.moreno@csic.es; 4 Department of Immunohematology and Blood Transfusion, Leiden University Medical Centre, 2333 ZA Leiden, The Netherlands; f.koning@lumc.nl

**Keywords:** celiac disease, HLA-DQ2.5, HLA-DQ8.1, molecular docking, quantitative matrix, peptide binding prediction

## Abstract

Human leukocyte antigens (HLAs) are pivotal in antigen processing, presenting to CD4+ T cells, and are linked to autoimmune disease susceptibility. In celiac disease, HLA-DQ2.5 and HLA-DQ8.1 bind gluten peptides on APCs, some recognized by CD4+ T cells, prompting inflammation and tissue damage. While extensively studied experimentally, these alleles lack comprehensive in silico analysis. To explore peptide–HLA preferences, we used molecular docking on peptide libraries, deriving quantitative matrices (QMs) for evaluating amino acids at nine-residue peptide binding cores. Our findings tie specific residue preferences to peptide backbone conformations. Validating QMs on known binders and non-binders showed strong predictive power (89–94% accuracy). These QMs excel in screening protein libraries, even whole proteomes, notably reducing time and costs for celiac disease risk assessment in novel proteins. This computational approach aligns with European Food Safety Authority guidance, promising efficient screening for potential celiac disease triggers.

## 1. Introduction

Human leukocyte antigens (HLAs) are major histocompatibility complex (MHC) proteins and are involved in antigen processing. Some of the antigens, synthesized in the cell or endocytosed and cleaved into oligopeptides, bind to a specific MHC protein [1]. Different peptides bind to different MHCs. The peptide–MHC complex is transferred to the cell membrane, where is recognized by specific CD4+ T cells and an immune response is initiated. 

HLA proteins are the most polymorphic proteins in higher vertebrates. They are divided into class I and class II. More than 25,000 alleles are known for class I and around 10,000 alleles for class II [2]. The HLA proteins of class II consist of two chains: α and β. The peptide binding site is formed by two antiparallel helices and two beta strands. It is open at both ends and accommodates peptides of different lengths, usually between 11 and 25 amino acids. The binding core itself consists of only nine residues. The rest of the residues flank both ends. The HLA class II proteins are grouped in three loci: DR, DQ, and DP. Some of them are associated with susceptibility to autoimmune diseases. For example, DRB1*04:01 is associated with susceptibility to rheumatoid arthritis [3,4], DRB1*03:01 to systematic lupus erythematosus [5,6], DQ8 (DQA1*0301, DQB1*0302) to type 1 diabetes [7,8], DPB1*0201 to chronic beryllium disease [9], etc.

Among the HLA-DQ proteins, DQ2.5 (DQA*05:01, DQB*02:01) and DQ8.1 (DQA*03:01, DQB*03:02) are associated with susceptibility to celiac disease [10,11]. Celiac disease is an autoimmune disorder triggered by gluten ingestion in genetically predisposed individuals. Gluten proteins undergo partial digestion within the human intestine, yielding numerous gluten peptides. These peptides cross the epithelial layer of the mucosa in the small intestine, reaching the lamina propria. Here, an enzyme known as transglutaminase 2 (TG2 or tTG2) catalyses their deamidation. This alteration increases their affinity to HLA-DQ2.5 and HLA-DQ8.1 proteins found on APCs. Subsequently, some of these peptides, when presented on the surface of APCs, are recognized by CD4+ T cells, thereby initiating inflammatory reactions and resulting in tissue damage [12,13]. Symptoms of celiac disease include diarrhoea, abdominal pain, bloating, and weight loss [14]. If left untreated, it can lead to malabsorption of nutrients and various complications. Diagnosis involves blood tests for specific antibodies and confirmatory intestinal biopsy [15]. The treatment requires a strict lifelong gluten-free diet, eliminating wheat, barley, and rye from the patient’s diet. Adherence to this diet helps alleviate symptoms and promotes intestinal healing [16]. DQ2.5 is present in 25% of the world population, while DQ8.1 is present in 14% [17]. Only 3% of the individuals carrying any of these two alleles develop celiac disease [18]. However, among the patients with celiac disease, 80–85% carry only DQ2.5, 5–11% carry only DQ8.1, and 3–10% carry both [10,11].

One of the possible reasons for making these proteins susceptible to celiac disease is the structure of one of the pockets in the binding site, i.e., pocket 9. In DQ2.5 and DQ8.1, the Asp^57β^ is mutated to Ala. In most HLA class II proteins, Asp^57β^ forms two salt bridges with Arg^76α^. These interactions neutralize the charge in this pocket. The mutation of Asp to Ala makes the pocket positively charged, as Ala is not able to form a salt bridge with Arg. Thus, peptides carrying a negatively charged Glu at p9 fit well into pocket 9 and make stable complexes with DQ2.5 and DQ8.1 [10].

Prior studies on the prediction of peptide binding on both alleles via different in silico tools were performed [19,20,21,22,23,24,25,26,27]. Costantini et al. applied homology modelling to model DQ2 and DQ8, and then the modelled complexes with known epitopes were subject to mild energy minimization [22]. The energy of the complexes was assessed via the docking module of the InsightII package, and intermolecular interactions were analysed [22]. Recently, in another study, molecular docking was applied on 26 known immunotoxic peptides to both DQ alleles using the automatic GRAMM-X online server [25]. In our previous study, we applied flexible peptide sidechain molecular docking to derive models for prediction of peptide binding to different HLA-DQ alleles, including HLA-DQ2.5 [27]. The models were implemented via the server EpiDock (http://www.ddg-pharmfac.net/epidock/, accessed on 29 November 2023).

Here, we explore the amino acid preferences in peptides that bind to HLA-DQ2.5 and HLA-DQ8.1 through the application of full flexible binding site molecular docking using combinatorial peptide libraries. Each peptide from the libraries underwent docking into the peptide binding site, and the resulting binding scores were normalized to evaluate the amino acid contributions at each peptide position. These contributions effectively differentiated between preferred and non-preferred residues.

To examine the impact of the peptide backbone on the preferences for specific residues, we simulated the binding modes of two peptides targeting DQ2.5 by molecular dynamics. One of the peptides was the α-gliadin peptide QPFPQPELPYP, characterized by a conformationally rigid backbone due to the presence of five Pro residues. The other peptide was the more flexible non-gliadin binder WIEQEGPEYWD. Our findings reveal that the peptide backbone conformation exerts a significant influence on both the binding mode and the preferences for specific residues at certain positions within the peptide binding core.

## 2. Results

### 2.1. Docking-Based Quantitative Matrices (QMs) for Prediction of Peptide Binding to HLA-DQ2.5

The X-ray structure of HLA-DQ2.5 in complex with the rigid α-gliadin peptide Q_0_P_1_F_2_P_3_Q_4_P_5_E_6_L_7_P_8_Y_9_P_10_ (pdb code: 6mfg) [28] was used in the molecular docking calculations and molecular dynamics (MD) simulations. The more flexible non-gliadin binding endecamer W_0_I_1_E_2_Q_3_E_4_G_5_P_6_E_7_Y_8_W_9_D_10_ [29], originating from HLA class I α-chain (UniProt: A0A0G2JL56), was modelled by homology using the 6mfg complex as a starting conformation. 

**Figure 1:**
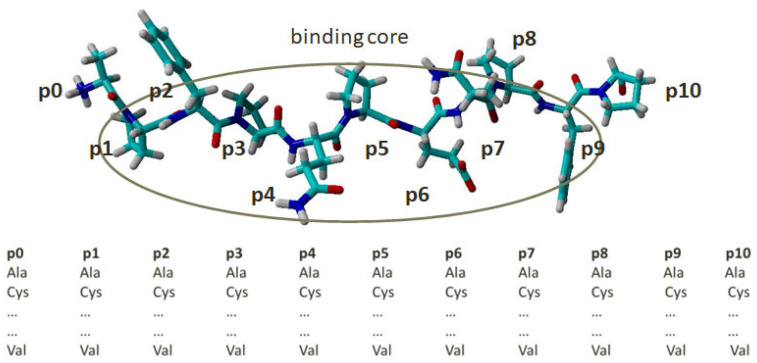
Combinatorial peptide library constructed by single amino acid substitution (SAAS). Positions from 1 to 9 denote the peptide binding core. Positions 0 and 10 are flanking residues. The residue at each position is substituted for by the remaining 19 naturally occurring amino acids. The atoms are coloured by element—carbon in cyan, oxyden in red, nitrogen in blue, and hydrogen in white.

The bound peptide in each complex was used as a parent peptide for the construction of a combinatorial peptide library by single amino acid substitution (SAAS), i.e., a residue at a given position in the parent peptide was substituted for with the remaining 19 naturally occurring amino acids—one substitution produces one peptide (Figure 1). The peptide binding core includes the residues from p1 to p9. Next to them at both ends are the flanking residues p0 and p10. The library contains 210 endecamers (19 amino acids × 11 positions + 1 parent peptide). 

Each peptide from the library was docked into the peptide binding site on HLA-DQ2.5 following the protocol described in Section 4. The protocol was carefully optimized, considering the flexibility of the peptide and binding site, exhaustiveness, and energy range parameters. To generate quantitative matrices (QMs), the binding scores, assigned as affinities, were normalized over both the binding core and the entire peptide. As a result, two QMs were created for each set of parameters, specifically for nonamers and endecamers. The predictive performance of the QMs was evaluated using external test sets, and the QM with the highest *sensitivity*, *specificity*, and *accuracy* was chosen for further analysis.

For the α-gliadin peptide library, the best-performing QM was obtained using the following settings: flexible peptide and binding site, exhaustiveness of 8, energy range of 3 kcal/mol, and normalization over the binding core (Table S1). This QM successfully identified 95% of the binding peptides from the test set and 83% of the non-binders at cutoff 0.1 (Table 1, and Figure S1a). Similarly, for the non-gliadin peptide library, the optimal QM settings were the following: flexible peptide and binding site, exhaustiveness of 8, energy range of 3 kcal/mol, and normalization over the binding core (Table S2). This QM achieved a recognition rate of 93% for the test binders and 89% for the non-binders at cutoff 0.2 (Table 1 and Figure S1a). In both QMs, positive coefficients indicate positive contributions to the peptides’ affinity for the HLA-DQ2.5 protein, while negative coefficients represent negative contributions.

**Figure 2:**
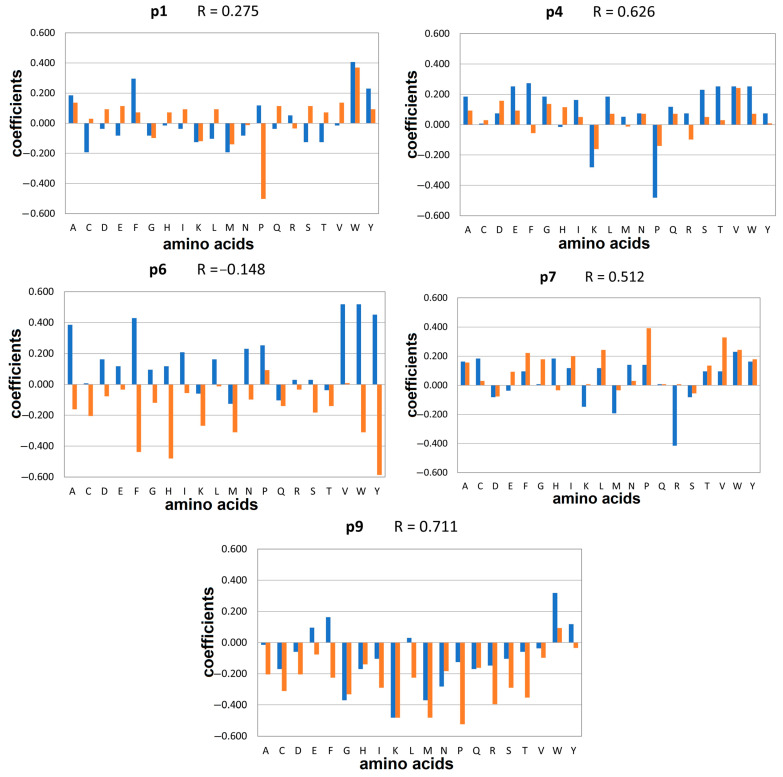
Contributions of the amino acids at the anchor positions p1, p4, p6, p7, and p9 according to the QMs’ coefficients for α-gliadin (blue bars) and non-gliadin peptide libraries (orange bars) for HLA-DQ2.5. The correlation coefficients between the contributions are given in the chart titles. Positive coefficients indicate positive contributions and negative coefficients indicate negative contributions.

Within the peptide binding core, specific anchor positions are responsible for binding to protein pockets with distinct structures and polarity. For HLA-DQ2.5, these anchor positions include p1, p4, p6, and/or p7 and p9 [30]. The preferred amino acids at these anchor positions for the α-gliadin and non-gliadin peptide libraries, as determined by the QMs, are shown in Figure 2. The correlation coefficients range from −0.148 for p6 to 0.711 for p9. This suggests that the preferences for specific residues at the anchor positions are strongly influenced by the backbone conformation.

In the α-gliadin library, the substitution of Pro at p1 with Trp, Phe, Tyr, or Ala enhanced the docking affinity. In the non-gliadin library, replacing Ile with Trp, Ala, Val, Glu, Gln, or Ser led to an increase in predicted affinity. However, the substitution of Ile with Pro at p1 in non-gliadins significantly decreased the affinity. Regarding p4, the preferences in the gliadin library favoured Phe, Trp, Thr, Val, and Glu, while among the non-gliadins, Val, Asp, and Gly were favoured. Pro and Lys were unfavourable in both libraries. The most significant differences between the gliadin and non-gliadin libraries were observed in the preferred and non-preferred amino acids at p6, as indicated by the negative correlation coefficient (R = −0.148). Replacing Glu in α-gliadin library with aromatic and aliphatic residues resulted in an increase in the binding scores. Conversely, in the non-gliadin library, substituting Pro for aromatic residues led to a decrease in affinity. At p7 in the gliadin library, a diverse range of amino acids including Trp, His, and Cys were well tolerated, while the presence of Arg was unfavourable. In contrast, non-gliadins showed a preference for hydrophobic amino acids here without any significant disfavour towards specific residues. At p9, replacing Tyr with other aromatic amino acids such as Trp and Phe enhanced the affinity of gliadin peptides. For the non-gliadins, Trp was the only preferred residue at p9, while Pro was the least preferred one.

Peptide–HLA interactions at the anchor positions within the peptide binding site are summarized in Table 2 and visually depicted in Figure S2. The binding pockets are denoted by a capital letter ‘P’ followed by a number corresponding to the peptide binding position. Trp emerges as a pivotal residue, exerting a positive influence on the affinity of α-gliadin peptides binding to HLA-DQ2.5. Trp was notably favoured at positions p1, p6, p7, and p9, where it established hydrogen bonds with Asn^62α^ when occupying pockets P6, P7, or P9. Furthermore, Trp was engaged in π-π stacking interactions with Phe^53α^, Phe^11β^, Phe^47β^, and Trp^61β^, contributing significantly to peptide binding. Additionally, it participated in hydrophobic interactions with multiple amino acids across both α and β HLA chains. Phe was the prominent residue in pocket P4, forming two hydrogen bonds with Tyr^9α^ and Asn^62α^ and demonstrating hydrophobic interactions with Phe^11β^, Leu^26β^, and Lys^71β^. Among the non-preferred residues, Met in pockets P1 and P6 had a detrimental effect on binding, while Pro in pocket P4, Arg in pocket P7, and Lys in pocket P9 also yielded unfavourable outcomes. Arg from p7 formed an intramolecular hydrogen bond with Gln at p6, evidently disturbing the binding. The negative effect of Lys at p9 can be attributed to the presence of the positively charged Arg^76α^ in pocket P9 [10].

In the non-gliadin library, Trp emerged as the most preferred residue in pockets P1 and P9, while Pro was the most favoured in pockets P6 and P7, and Val was the most preferred choice in P4 (Table 2, Figure S2). Notably, Tyr^9α^, Arg^53α^, and Asn^69α^ were engaged in hydrogen bonding with the peptide residues at positions p2, p1, p7, and p9, respectively. Additionally, Trp at p1 formed π-π stacking interactions with Phe^33α^, Phe^52α^, and Phe^54α^. Conversely, Pro was the least preferred at positions p1 and p9, Lys was most unfavourable at p4, Tyr was least preferred at p6, and Asp was the least preferred at p7.

### 2.2. MD Simulations of Peptides Carrying Trp at p10

**Figure 3:**
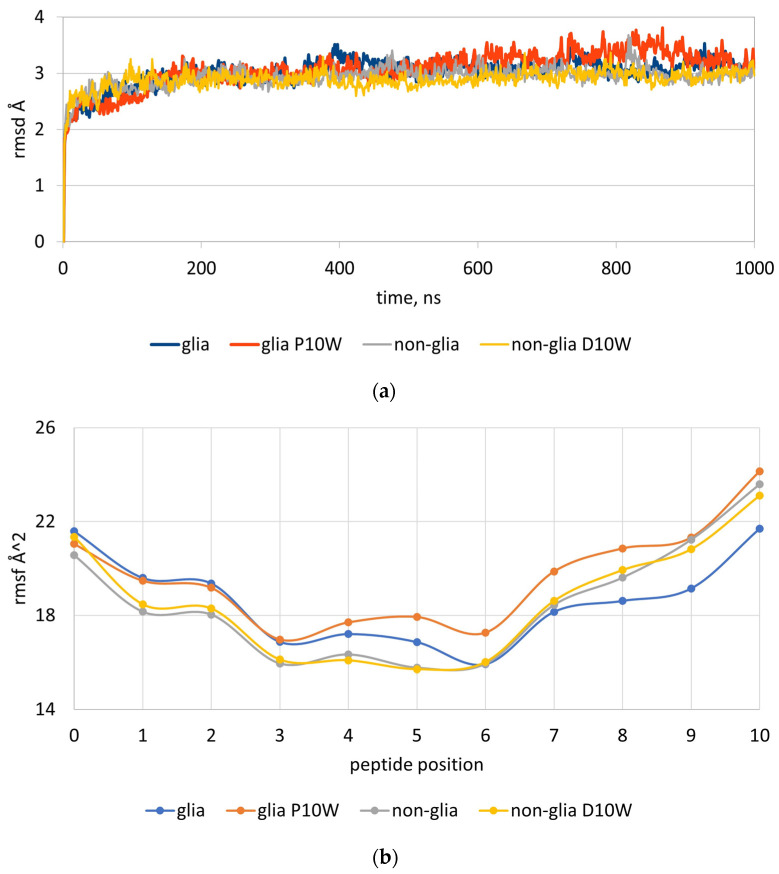

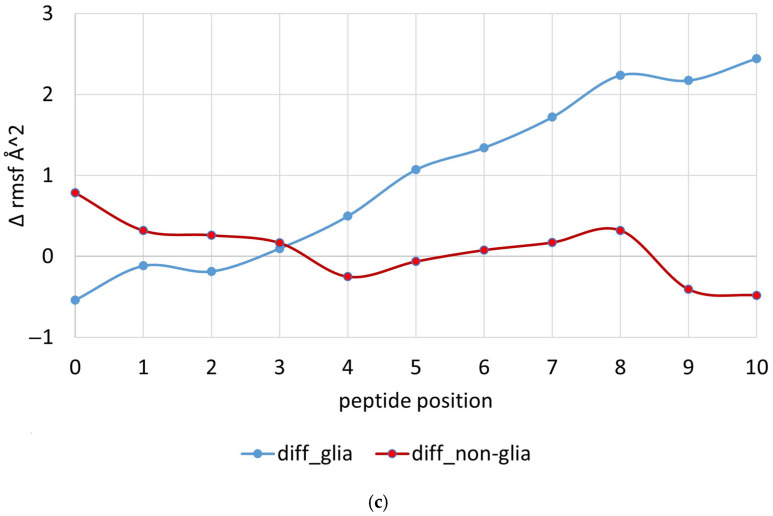
Root-mean-square deviations (rmsds) (**a**), root-mean-square fluctuations (rmsfs) (**b**), and differences in the root-mean-square fluctuations (Δrmsfs) (**c**) of peptides’ Cα atoms. In (**a**,**b**), α-gliadin peptide (QPFPQPELPYP) is shown in blue, α-gliadin peptide with P10W mutation (QPFPQPELP-YW) in orange, non-gliadin peptide (WIEQEGPEYWD) in grey, and non-gliadin peptide with D10W mutation (WIEQEGPEYWW) in yellow. In (**c**), rmsd differences between the wild and mutant α-gliadin peptides (blue curve) and non-gliadin peptides (red curve) are depicted.

In the gliadin peptide library, among the non-preferred amino acids for the flanking residues p0 and p10, the highest negative contribution belongs to Trp at p10. The value for Trp10 is −0.649. In the non-gliadin peptide library, this value is 0.136. As no information about the detrimental contribution of Trp at p10 was available in the literature, we examined the effect of Trp10 on the stability of the peptide–HLA complexes by MD simulations on four peptides: α-gliadin peptide QPFPQPELPYP, α-gliadin peptide with P10W mutation QPFPQPELPYW, non-gliadin peptide WIEQEGPEYWD, and non-gliadin peptide with D10W mutation WIEQEGPEYWW. The complexes were simulated for 1000 ns and the root-mean-square deviations of atomic coordinates over time (rmsds) and the root-mean-square fluctuations (rmsfs) of Cα were recorded at each peptide position per ns. Three runs were performed for each complex. The average rmsd, rmsf, and differences in the fluctuations (Δrmsfs) between the wild and mutant peptides are given in Figure 3. 

The rmsds of the four complexes reached a stationary shape before 100 ns (Figure 3a). Greater deviations were observed for α-gliadin peptide with P10W mutation (Figure 3a). It is evident that the presence of Trp at p10 in the rigid gliadin peptide causes a huge distortion not only on p10 but also on p5, p6, p7, p8, and p9 positions (Figure 3b). The differences in rmsf for p5–p10 were between 1.07 and 2.44 Å^2^ (Figure 3c). No distortion was observed in the more flexible non-gliadin peptide when Trp was present at p10. The differences here were between −0.06 and −0.48 Å^2^. 

### 2.3. Docking-Based Quantitative Matrices (QMs) for Prediction of Peptide Binding to HLA-DQ8.1

The X-ray structure of HLA-DQ8.1 in complex with the T-cell epitope from α-gliadin S-_1_G_0_E_1_G_2_S_3_F_4_Q_5_P_6_S_7_Q_8_E_9_N_10_P_11_ (pdb code: 2nna) [10] served as the basis for the molecular docking studies. The bound peptide parented a combinatorial library consisting of 210 endecamers. Docking simulations were performed using various settings, and the best predictions were obtained with a flexible peptide and protein binding site, an exhaustiveness of 8, an energy range of 3 kcal/mol, and normalization over the binding core (Table S3). The resulting QM achieved a recognition rate of 94% for both binding and non-binding peptides in the external test set at a cutoff of 0.3 (Table 1 and Figure S1b). 

**Figure 4:**
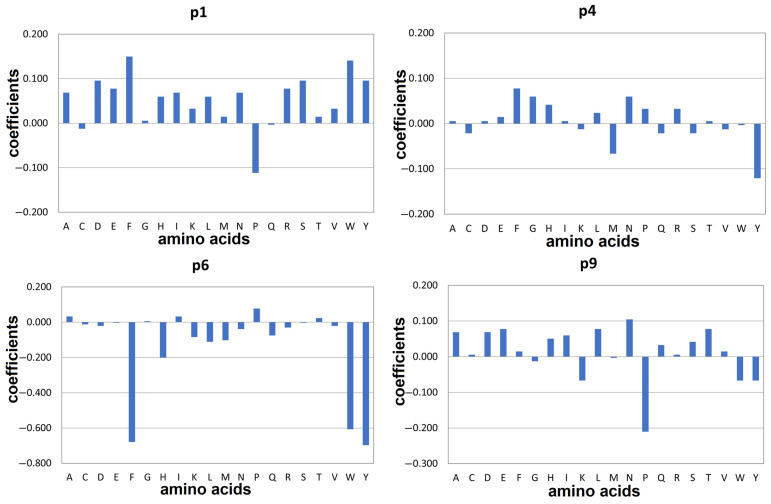
Contributions of the amino acids at anchor positions p1, p4, p6, and p9 according to the QM for α-gliadin peptide library for HLA-DQ8.1. Positive coefficients indicate positive contributions and negative coefficients indicate negative contributions.

Figure 4 displays the bar charts illustrating the preferences and non-preferences of residues at anchor positions p1, p4, p6, and p9 for peptide binding to HLA-DQ8.1 [7]. At p1, Phe and Trp were the most preferred residues establishing hydrogen bonds with Arg^53α^ (Table 2, Figure S2). Pro was not favoured at this position. At p4, Phe was the most favoured, while Tyr was the least preferred. Both Phe and Tyr were involved in hydrogen bonds with Tyr^9α^ and Asn^62α^. However, the hydroxyl group of Tyr can lead to clashes with Val^78β^ (Figure S2). At p6, Pro emerged as the most favoured residue, while aromatic residues such as Tyr, Phe, and Trp had detrimental effects, with contributions ranging from −0.7 to −0.6. For p9, the preferences indicate that Asn, Glu, Thr, and Leu are the favoured residues. For example, Asn formed two hydrogen bonds with His^68α^ and Asn^69α^ from pocket P9 (Table 2, Figure S2). Conversely, Pro was the least favoured residue at this position.

## 3. Discussion

The European Food Safety Authority (EFSA) is responsible for assessing and providing scientific advice on food safety and nutrition-related issues within the European Union. To this end, EFSA plays a crucial role in ensuring the safety of novel food products for individuals with celiac disease [31]. Recently, EFSA has issued guidance on assessing the risk of novel proteins causing celiac disease [32]. The guidance defines a framework that utilizes various methods, such as in silico, in vitro, and in vivo testing, to evaluate the potential of new proteins to trigger an immune response. The novel protein is initially compared to known proteins associated with celiac disease, and if any relevant identities and/or similarities are found, the protein is searched for binders to HLA-DQ alleles associated with celiac disease. If strong peptide binders are predicted, HLA-DQ binding assays, in vitro digestibility tests, and tests for T-cell epitopes are conducted to confirm or reject these predictions. This guidance ensures the safety of novel proteins and assists food manufacturers and regulatory agencies in assessing the risk of celiac disease.

Here, following the principles in EFSA’s guidance for risk assessment of novel proteins, we describe the development of three docking-based models for the prediction of peptide binding to two HLA-DQ alleles associated with susceptibility to celiac disease. HLA-DQ2.5 is the main such allele available in 80–85% of the patients diagnosed with celiac disease [12]. The peptide binding motif for HLA-DQ2.5 includes preferences for aromatic or hydrophobic aliphatic residues in p1, acidic residues in p4, p6, and/or p7, and hydrophobic or aromatic residues at p9 [24,33]. Additionally, Stepniak et al. [34] found that the T-cell epitopes originating from HLA-DQ2.5 binders preferably carry Pro at p1, p3 and p8 and Glu at p4 and p6. Based on high-throughput mass spectroscopy of HLA ligand elution data, Kosaloglu-Yalcın et al. identified three binding motifs for HLA-DQ2.5: ‘motif 1’ (Glu and Asp preferred at p4, p6, and p7), ‘motif 2’ (Pro at p1, Glu at p7 and p9), and ‘no clear motif’ (hydrophobic residues at p1, p4, and p6, Glu at p7 and p9) [30]. All these findings indicate that peptides bind to HLA-DQ2.5 in diverse modes, leading to varying preferences for specific residues at anchor positions. 

**Figure 5:**
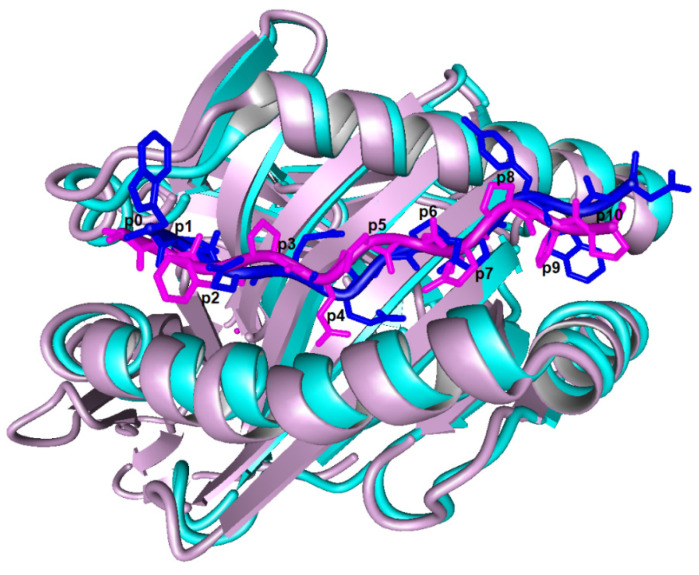
Superposition of α-gliadin peptide QPFPQPELPYP (magenta), HLA-DQ2.5 (mauve), and non-gliadin peptide WIEQEGPEYWD (blue) HLA-DQ2.5 (cyan). The rmsd over peptide backbones is 2.460 Å.

Our studies revealed that amino acid preferences are influenced by the peptide backbone conformation. The superposition of the gliadin and non-gliadin peptides shows that the non-gliadin peptide has a more deeply buried p4 and a more flanking p10 (Figure 5). The presence of a deeply buried p4 explains the preference for small amino acids such as Val, Asp, and Gly at this position (Table S2). On the other hand, the greater freedom at p10 justifies the acceptance of bulky amino acids like Trp, which is not tolerated at the same position in the gliadin peptide due to the rigid poly-Pro backbone, as we demonstrated by MD simulations.

The results of our study align well with the known binding motifs for HLA-DQ2.5 [30], indicating that most of the preferred and non-preferred amino acids are in agreement. Both gliadin and non-gliadin libraries accept aromatic and aliphatic amino acids at p1. However, the presence of Pro at p1 has different effects: it is preferred as part of the poly-Pro backbone in gliadins, while it is detrimental in non-gliadin sequences. Hydrophobic or acidic residues are favoured at p4 in both gliadins and non-gliadins. At p6, significant differences emerge between gliadins and non-gliadins concerning aromatic residues: gliadins prefer them, whereas non-gliadins tend to avoid them. One possible explanation for these differences could be the presence of Pro, this time in the non-gliadin peptide. Substituting the rigid Pro at p6 with bulky aromatic residues may cause unfavourable distortion in the peptide backbone. Hydrophobic amino acids are well-suited for pocket 7. There is a consensus regarding the preferred aromatic amino acids and Glu at p9 between the gliadin and non-gliadin libraries, which aligns with Kosaloglu-Yalcın’s observation of ‘no clear motif’ [30].

The peptide binding motif for HLA-DQ8.1 includes negatively charged amino acids at both p1 and p9, bulky but not basic residues at p4, and small residues at p6 [19,35,36,37,38]. The preferences and non-preferences identified in the present study by docking simulations fully agree with the known binding motif. Additionally, our findings reveal that Phe and Tyr also are well accepted at p1, Pro at p1 and p9, Lys at p4; Phe, Tyr, Arg, and His are unfavoured.

The QMs derived in the present study by docking simulations of combinatorial peptide libraries enable the screening of vast protein libraries, even whole proteomes, significantly reducing time and cost compared to experimental methods. Moreover, they facilitate the exploration of various binding conformations and the prediction of binding affinities. The presented QMs will be implemented in a software tool for predicting peptide binding to alleles associated with celiac disease. In line with the principles in the EFSA’s guidance for allergenicity risk assessment of novel proteins, this implementation will enhance the predictive capabilities and aid in the assessment of potential risks. 

## 4. Materials and Methods

### 4.1. Structures and Combinatorial Libraries

The X-ray structure of HLA-DQ2.5 in complex with the α-gliadin peptide Q_0_P_1_F_2_P_3_Q_4_P_5_E_6_L_7_P_8_Y_9_P_10_ (pdb code: 6mfg [28]) was used in the molecular docking calculations and MD simulations. The non-gliadin endecamer peptide W_0_I_1_E_2_Q_3_E_4_G_5_P_6_E_7_Y_8_W_9_D_10_ [29] was modelled by homology using 6mfg as a starting conformation and geometry optimized by MD simulation for 1000 ns. The crystallographic structure of the complex between HLA-DQ8.1 and α-gliadin peptide S-_1_G_0_E_1_G_2_S_3_F_4_Q_5_P_6_S_7_Q_8_E_9_N_10_P_11_ (pdb code: 2nna [10]) was used. 

The peptides bound in HLA-DQ parented combinatorial libraries constructed by the method of single amino acid substitution, i.e., the residues at each position in the parent peptide were substituted for with the remaining 19 naturally occurring amino acids following the rule ‘one peptide–one substitution’. Each library consisted of 210 endecamers (19 amino acids × 11 positions + 1 parent peptide) and covered the binding core plus the flanking p0 and p10. Positions p−1 and p11 in the peptides for DQ8.1 were kept unchanged. 

The external test set used for the validation of the derived QMs for HLA-DQ2.5 consisted of 4249 binders and 4249 non-binders. The DQ2.5 binders were qualitatively measured by mass spectrometry [39]. Only peptides longer than 9 amino acids were included in the set. The set of non-binders was generated by a random combination of non-preferred amino acids at all nine positions from the binding core [30]. 

The test set for validation of the QMs for HLA-DQ8.1 was constructed similarly to the test set for HLA-DQ2.5. A set of 4339 strong binding nonamers eluted by LC-MS was selected as a positive test set [7]. The same number of non-binders was generated by random combination of non-preferred amino acids in the binding core according to the binding motif for HLA-DQ8.1 [7]. 

The binding affinities of the tested peptides were calculated as a sum of the amino acids’ contributions at each binding core position. The accuracy of predictions was assessed at different cutoffs between binders and non-binders in the range between −0.5 and +0.5 with a step of 0.1, and the cutoff with the highest accuracy was selected. 

### 4.2. Molecular Docking Protocol

Proteins and peptides were pre-processed by MGLTools v. 1.5.7 (Scripps, San Diego, CA, USA). Mutations and visualizations were performed by YASARA (YASARA Biosciences GmbH, Wien, Austria). Each peptide from the combinatorial libraries was docked into the corresponding HLA-DQ protein using the software tool AutoDock Vina v. 1.2.0 [40]. The docking was performed using the AutoDock Vina forcefield, and the resulting binding energy was reported as an affinity in kcal/mol. To optimize the docking protocols, several parameters were considered, including the flexibility of the peptide and protein binding site, exhaustiveness, and energy range. The binding sites were defined to include protein residues within a 6 Å distance from the bound peptide. AutoDock Vina provided the exhaustiveness parameter, which determines the computational effort used during a docking experiment [41]. The default exhaustiveness value is 8, but in this study, exhaustiveness was tested by increasing it to 30, resulting in more consistent docking results. The energy range parameter defines the range of the best-scored poses [41]. Binding poses with scores outside this range were discarded. In this study, different energy ranges were tested, ranging from 1 to 6 kcal/mol. The best scores obtained for each peptide in the library were mean-normalized, and a quantitative matrix (QM) was constructed. To validate the QMs, external test sets were used, and the best-performing QMs were selected for further analysis.

### 4.3. MD Simulations Protocol

Initially, the energy of the peptide–HLA complexes was minimized for 10,000 steps using 10 kcal/molÅ^2^ harmonic restraints on solute-heavy atoms and a 10 Å cutoff for the non-bonded van der Waals and electrostatic interactions. Next, the complexes were heated to 300 K in a timestep of 2 fs for 100 ps, density-equilibrated for 100 ps, and equilibrated for 1 ns at constant temperature (300 K) and constant pressure (1 bar). Finally, the production phase was run for 1000 ns (1 μs). Frames were recorded every 1 ns for a total of 1000 per trajectory. The MD simulations in the present study were performed using AMBER 18 [42]. The trajectories were analysed by cpptraj V4.24.0 [43]. Two parameters were calculated: root-mean-square deviation (rmsd), which accounts for changes in atomic coordinates of input frames to a reference frame (frame #1 by default) and rmsfs (root-mean-square fluctuations), which account for atomic positional fluctuations. Three runs were conducted for each complex.

## 5. Conclusions

The EFSA’s role in ensuring food safety, especially for individuals with celiac disease, is evident in their guidance for assessing the risk of novel proteins causing immune reactions linked to celiac disease. Their comprehensive framework, using in silico, in vitro, and in vivo methods, is pivotal in evaluating these risks, requiring comparison to known celiac-associated proteins and subsequent binding assays.

The results found in this study represent a pivotal step in applying the EFSA’s risk assessment principles for novel proteins. The derived docking-based models (QMs) for peptide binding to HLA-DQ alleles linked to celiac disease enhance our understanding of peptide–HLA interactions, reveal diverse binding modes, and link specific residue preferences to peptide backbone conformations. Validation of these QMs on known binders and non-binders displayed substantial predictive accuracy (89–94%). QMs demonstrate their efficiency in screening protein libraries, including whole proteomes, significantly reducing time and cost compared to experimental methods. These QMs will be integrated into software for predicting peptide binding, aligning with the EFSA’s principles and enhancing risk assessment for proteins triggering celiac disease. 

## Figures and Tables

**Table 1:** Predictive ability of the QMs generated on the peptide-based combinatorial libraries docked on HLA-DQ2.5 and HLA-DQ8.1. *Sensitivity* accounts for the correct prediction of binders, *specificity* for the correct prediction of non-binders, *accuracy* for both.

QM	*Sensitivity*	*Specificity*	*Accuracy*	*Cutoff*
HLA-DQ2.5				
α-gliadin peptide library	95	83	89	0.1
non-gliadin peptide library	93	89	91	0.2
HLA-DQ8.1				
α-gliadin peptide library	94	94	94	0.3

**Table 2:** Intermolecular interactions between the most positively/negatively contributing peptide residues from α-gliadin and non-gliadin peptides binding into the corresponding pockets of HLA-DQ2.5 and HLA-DQ8.1. The residues of the corresponding peptide position are given in bold; hb stands for hydrogen bond, hpho for hydrophobic interaction; π-π–π-π contact.

Pocket	Contribution	DQ2.5		DQ8.1 α-Glia aa
		α-Glia aa	non-Glia aa	
P1	positive	**W**: π-π—F53α; hpho—W43α, F51α, F54α, N82β, L85β	**W**: hb—R53α; π-π—F33α; F52α, F54α; hpho—F33α, F52α, F54α, N82β, E86β	**F**: hb—R53α; hpho—Y9α, F54α, L85β
	negative	**M**: hpho—W43α, L85β	**P**: hpho—F54α	**P**: hpho—F54α
P4	positive	**F**: hb—Y9α, N62α; hpho—F11β, L26β, K71β	**V**: hb—Y9α; hpho—L26β, K71β, V78β	**F**: hb—Y9α, N62α; hpho—F11β, L26β, T28β, K71β, E74β
	negative	**P**: hpho—V78β	**K**: hb—Y9α, G10α; intramolecular hb with G5; hpho—L26β, K71β	**Y**: hb—Y9α, N62α; hpho—F11β, T28β, E74β, V78β
P6	positive	**W**: hb—N62α; π-π—F11β, W61β hpho—V65α, L66α, F11β, S30β, W61β	**P**: hpho—V65α, L66α, F11β	**P**: hpho—V65α, F11β, Y30β
	negative	**M**: hb—N62α; hpho—N62α,V65α, F11β	**Y**: hb—N62α; π-π—F11β, W61β hpho—V65α, Y9β, F11β	**Y**: π-π—F11β hpho—N62α,V65α, F11β
P7	positive	**W**: hb—N69α; π-π—F11β, F47β, W61β; hpho—F11β, S28β, F47β, W61β, K71β	**P**: hb—N69α; hpho—W61β, K71β	-
	negative	**R**: hb—N69α; intramolecular hb with Q6; hpho—F47β, W61β	**D**: hb—N69α, K71β; hpho—W61β	-
P9	positive	**W**: hb—N69α; hpho—S72α, L73α, I37β	**W**: hb—N69α; hpho—L73α, I37β, W61β	**N**: hb—H68α, N69α
	negative	**K**: hb—N69α; hpho—S72α	**P**: no contacts	**P**: hb—H68α; hpho—W61β

## Data Availability

Data are contained within the article and Supplementary Materials.

## Supplementary Materials

The following supporting information can be downloaded at: https://www.mdpi.com/article/10.3390/molecules29010138/s1, Table S1. Docking-based quantitative matrix for α-gliadin peptide library docked in HLA-DQ2.5; Table S2. Docking-based quantitative matrix for non-gliadin peptide library docked in HLA-DQ2.5; Table S3. Docking-based quantitative matrix for α-gliadin peptide library docked in HLA-DQ8.1. Figure S1a. Accuracy of predictions by the QMs for α-gliadin peptide (blue curve) and non-gliadin peptide (orange curve) at different cutoffs between binders and non-binders to HLA-DQ2.5. Figure S1b. Accuracy of predictions by the QM for HLA-DQ8.1 at different cutoffs between binders and non-binders. Figure S2. Intermolecular interactions between the most positively/negatively contributing peptide residues from α-gliadin and non-gliadin peptides binding into the corresponding pockets of HLA-DQ2.5 and HLA-DQ8.1. The hydrogen bonds are presented as orange discontinued lines; π-π-stacking with red or pale pink for weaker contacts, hydrophobic interactions with green or pale green for weaker interactions. The peptide backbone and sidechains are coloured in cyan. Hydrogens are not presented.
