# Supplementary material for: Assessment of Novel Proteins Triggering Celiac Disease via Docking-Based Approach
Source: Molecules. 2023 Dec 26;29(1):138. doi: 10.3390/molecules29010138 (PMC10780262; doi:10.3390/molecules29010138)
Supplement: Supplementary file 1 [file molecules-29-00138-s001.zip › molecules-2772907-supplementary.pdf]

molecules

MDPI

Article

Assessment of Novel Proteins Triggering Celiac Disease via Docking-Based Approach

Mariyana Atanasova [1,*], Ivan Dimitrov [1], Antonio Fernandez [2], Javier Moreno [3], Frits Koning [4] and Irini Doytchinova [1]

[1] Faculty of Pharmacy, Medical University of Sofia, 1000 Sofia, Bulgaria; idimitrov@pharmfac.mu-sofia.bg (I.D.); idoytchinova@pharmfac.mu-sofia.bg (I.D.)
[2] European Food Safety Authority, 43126 Parma, Italy; antonio.fernandezdumont@efsa.europa.eu
[3] Instituto de Investigación en Ciencias de la Alimentación (CIAL), Consejo Superior de Investigaciones Cientificas-Universidad Autonoma de Madrid (CSIC-UAM), Campus of Interntional Excellence—CEI (UAM+CSIC), Nicolás Cabrera, 9, 28049 Madrid, Spain; javier.moreno@csic.es
[4] Department of Immunohematology and Blood Transfusion, Leiden University Medical Centre, 2333 ZA Leiden, The Netherlands; f.koning@lumc.nl
* Correspondence: matanasova@pharmfac.mu-sofia.bg

Supplementary Materials:

Table S1. Docking-based quantitative matrix for α-gliadin peptide library docked in HLA-DQ2.5

aa	p1	p2	p3	p4	p5	p6	p7	p8	p9
A	0.185	0.207	0.030	0.185	-0.081	0.385	0.163	-0.104	-0.015
C	-0.193	0.074	-0.170	0.007	-0.170	0.007	0.185	-0.104	-0.170
D	-0.037	0.052	-0.148	0.074	-0.015	0.163	-0.081	-0.170	-0.059
E	-0.081	0.119	-0.081	0.252	-0.037	0.119	-0.037	-0.215	0.096
F	0.296	0.119	-0.059	0.274	0.030	0.430	0.096	0.030	0.163
G	-0.081	0.296	0.141	0.185	0.096	0.096	0.007	0.119	-0.370
H	-0.015	-0.015	-0.193	-0.015	-0.170	0.119	0.185	-0.015	-0.170
I	-0.037	0.096	-0.326	0.163	-0.104	0.207	0.119	-0.104	-0.104
K	-0.126	-0.437	-0.459	-0.281	-0.370	-0.059	-0.148	-0.348	-0.481
L	-0.104	0.007	-0.459	0.185	-0.148	0.163	0.119	-0.059	0.030
M	-0.193	-0.059	-0.348	0.052	-0.193	-0.126	-0.193	-0.193	-0.370
N	-0.081	0.163	-0.081	0.074	-0.148	0.230	0.141	0.007	-0.281
P	0.119	0.274	0.119	-0.481	0.119	0.252	0.141	0.119	-0.126
Q	-0.037	0.119	-0.393	0.119	-0.104	-0.104	0.007	-0.148	-0.170
R	0.052	0.163	-0.281	0.074	-0.215	0.030	-0.415	-0.281	-0.148
S	-0.126	0.119	-0.081	0.230	-0.193	0.030	-0.081	-0.081	-0.104
T	-0.126	0.185	0.052	0.252	0.007	-0.037	0.096	-0.015	-0.059
V	-0.015	0.207	0.052	0.252	-0.059	0.519	0.096	-0.237	-0.037
W	0.407	0.096	0.007	0.252	-0.015	0.519	0.230	0.141	0.319
Y	0.230	0.074	-0.037	0.074	-0.037	0.452	0.163	0.119	0.119

Table S2. Docking-based quantitative matrix for non-gliadin peptide library docked in HLA-DQ2.5

aa	p1	p2	p3	p4	p5	p6	p7	p8	p9
A	0.136	0.094	0.285	0.094	-0.013	-0.162	0.157	0.243	-0.204
C	0.030	0.094	-0.098	0.030	0.009	-0.204	0.030	0.094	-0.311
D	0.094	0.200	0.009	0.157	0.051	-0.077	-0.077	-0.140	-0.204
E	0.115	0.094	0.030	0.094	-0.077	-0.034	0.094	0.072	-0.077
F	0.072	0.157	0.115	-0.055	0.030	-0.438	0.221	0.136	-0.226
G	-0.098	0.285	0.157	0.136	0.094	-0.119	0.179	0.051	-0.332
H	0.072	0.051	0.157	0.115	0.009	-0.481	-0.034	-0.034	-0.140
I	0.094	0.221	0.200	0.051	-0.183	-0.055	0.200	0.094	-0.289
K	-0.119	-0.055	-0.013	-0.162	-0.247	-0.268	0.009	-0.162	-0.481
L	0.094	-0.055	0.221	0.072	0.051	-0.013	0.243	0.030	-0.226
M	-0.140	-0.162	0.051	-0.013	-0.055	-0.311	-0.034	0.030	-0.481
N	-0.013	0.094	0.051	0.072	0.094	-0.098	0.030	0.009	-0.183
P	-0.502	0.285	0.285	-0.140	0.051	0.094	0.391	0.115	-0.523
Q	0.115	0.200	-0.055	0.072	-0.013	-0.140	0.009	-0.140	-0.162
R	-0.034	-0.119	-0.055	-0.098	-0.247	-0.034	0.009	-0.098	-0.396
S	0.115	0.115	0.051	0.051	0.115	-0.183	-0.055	-0.140	-0.289
T	0.072	0.030	0.243	0.030	0.030	-0.140	0.136	-0.013	-0.353
V	0.136	0.136	0.009	0.243	-0.013	0.009	0.328	0.179	-0.098
W	0.370	0.157	0.115	0.072	0.051	-0.311	0.243	0.264	0.094
Y	0.094	0.094	0.179	0.009	-0.055	-0.587	0.179	0.094	-0.034

Table S3. Docking-based quantitative matrix for α-gliadin peptide library docked in HLA-DQ8.1

aa	p1	p2	p3	p4	p5	p6	p7	p8	p9
A	0.069	0.105	0.006	0.006	0.168	0.033	0.123	0.186	0.069
C	-0.012	-0.084	0.024	-0.021	-0.021	-0.012	-0.084	0.024	0.006
D	0.096	-0.166	0.042	0.006	0.087	-0.021	0.033	0.123	0.069
E	0.078	-0.193	0.078	0.015	0.078	-0.003	0.051	0.132	0.078
F	0.150	-0.778	0.087	0.078	0.177	-0.679	0.186	0.159	0.015
G	0.006	0.078	0.069	0.060	0.060	0.006	0.141	0.150	-0.012
H	0.060	-0.652	0.096	0.042	0.105	-0.202	0.141	0.150	0.051
I	0.069	-0.238	-0.057	0.006	0.051	0.033	0.069	0.060	0.060
K	0.033	-0.120	-0.039	-0.012	-0.039	-0.084	0.024	0.006	-0.066
L	0.060	-0.247	-0.129	0.024	0.114	-0.111	0.069	0.123	0.078
M	0.015	-0.247	-0.039	-0.066	0.078	-0.102	-0.066	-0.283	-0.003
N	0.069	-0.129	0.069	0.060	0.114	-0.039	0.042	-0.229	0.105
P	-0.111	0.015	0.195	0.033	0.042	0.078	0.195	0.195	-0.211
Q	-0.003	-0.202	0.042	-0.021	0.078	-0.075	0.006	0.078	0.033
R	0.078	-0.120	-0.003	0.033	0.078	-0.030	0.123	0.060	0.006
S	0.096	-0.057	0.078	-0.021	0.069	-0.003	0.078	0.096	0.042
T	0.015	-0.102	0.006	0.006	0.069	0.024	0.024	0.042	0.078
V	0.033	-0.048	0.123	-0.012	0.123	-0.021	0.033	0.159	0.015
W	0.141	-0.598	0.186	-0.003	0.096	-0.607	0.222	0.087	-0.066
Y	0.096	-0.643	0.096	-0.120	0.096	-0.697	0.168	0.123	-0.066

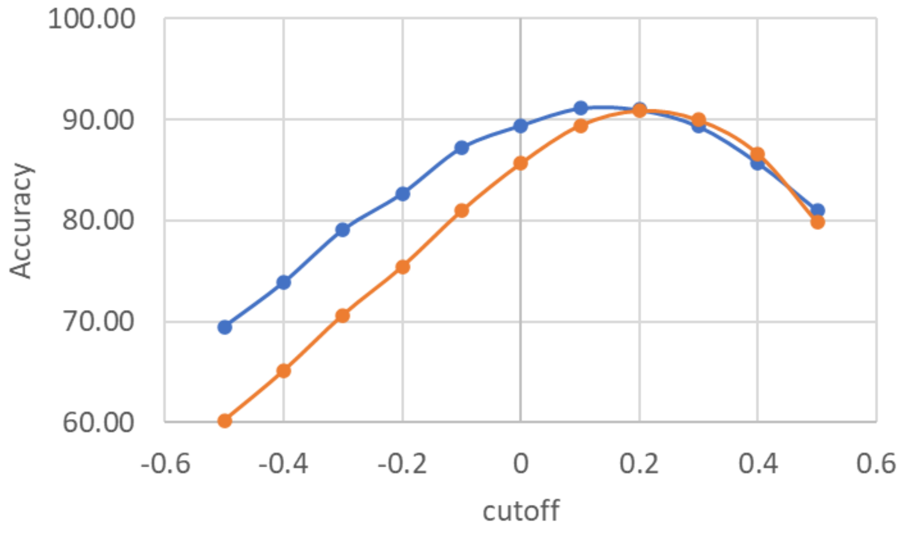

Figure S1a. Accuracy of predictions by the QMs for α-gliadin peptide (blue curve) and non-gliadin peptide (orange curve) at different cutoffs between binders and non-binders to HLA-DQ2.5.

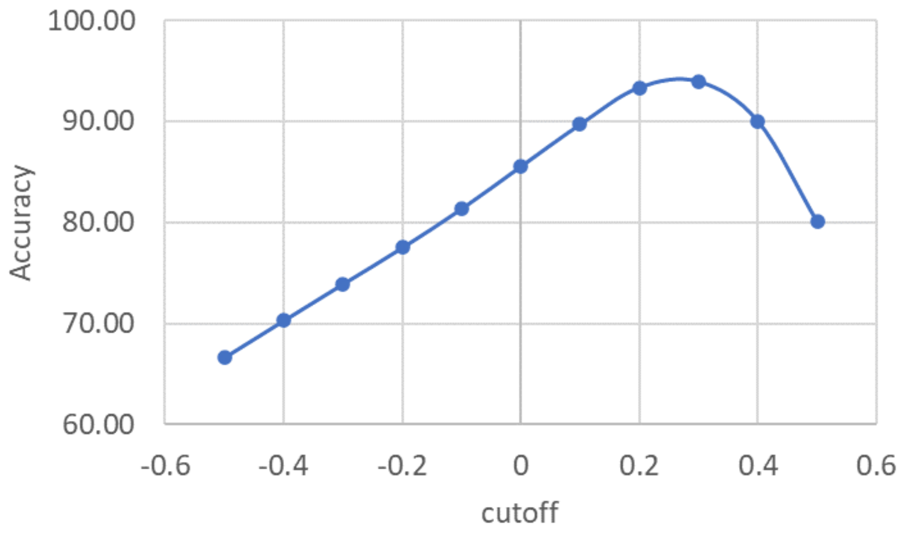

Figure S1b. Accuracy of predictions by the QM for HLA-DQ8.1 at different cutoffs between binders and non-binders.

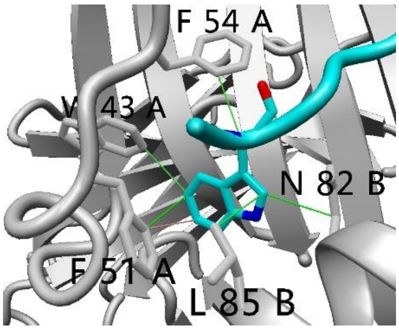

P1 positive: W α-glia DQ2.5

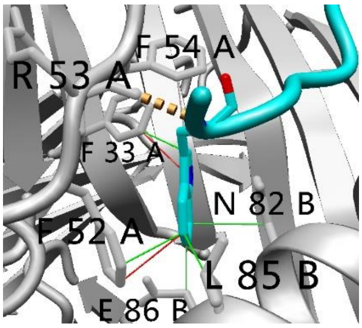

P1 positive: W non-glia DQ2.5

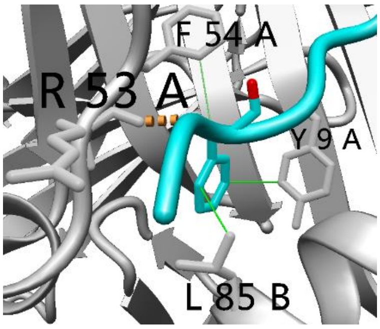

P1 positive: F α-glia DQ8.1

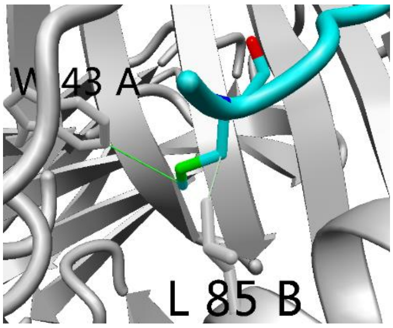

P1 negative: M α-glia DQ2.5

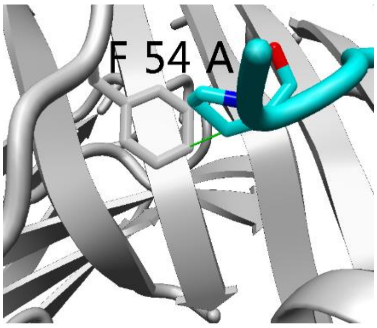

P1 negative: P non-glia DQ2.5

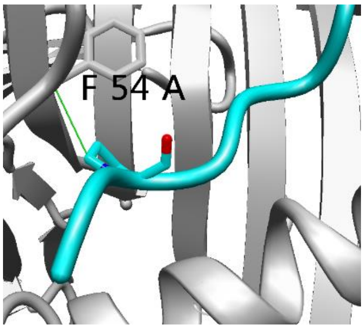

P1 negative: P α-glia DQ8.1

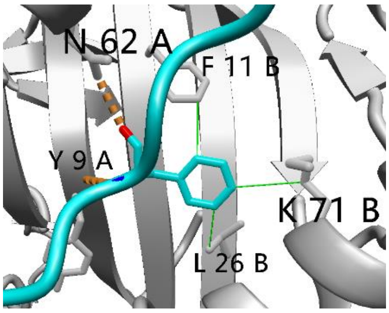

P4 positive: F α-glia DQ2.5

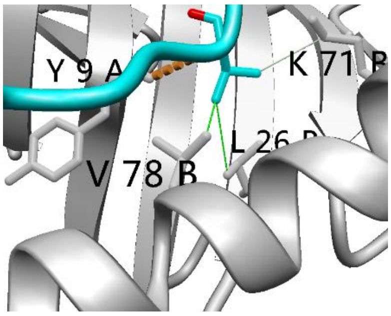

P4 positive: V non-glia DQ2.5

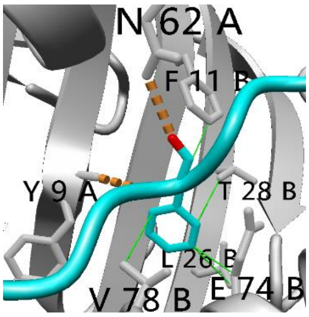

P4 positive: F α-glia DQ8.1

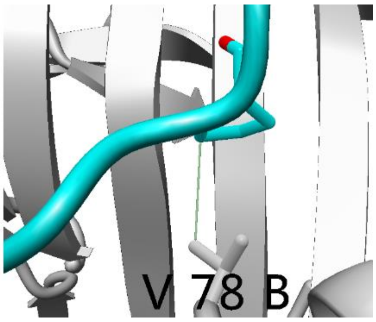

P4 negative: P α-glia DQ2.5

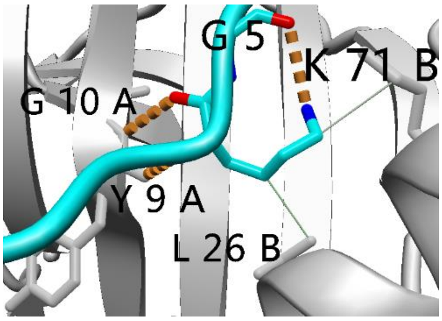

P4 negative: K non-glia DQ2.5

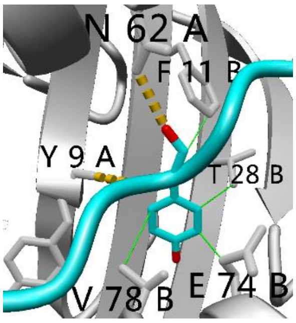

P4 negative: Y α-glia DQ8.1

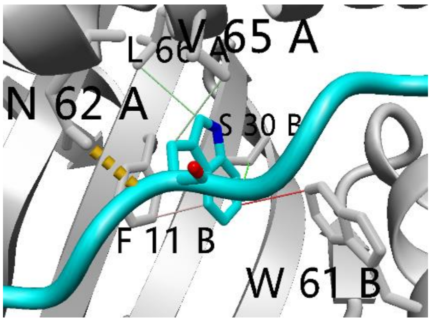

P6 positive: W α-glia DQ2.5

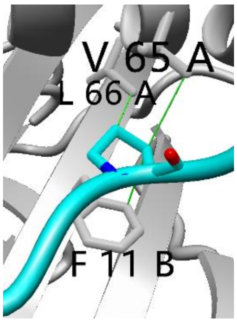

P6 positive: P non-glia DQ2.5

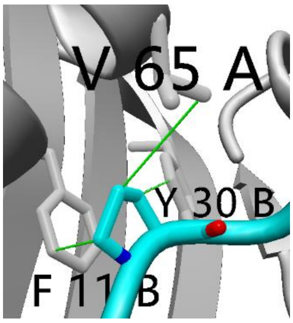

P6 positive: P α-glia DQ8.1

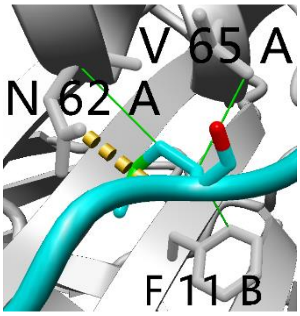

P6 negative: M α-glia DQ2.5

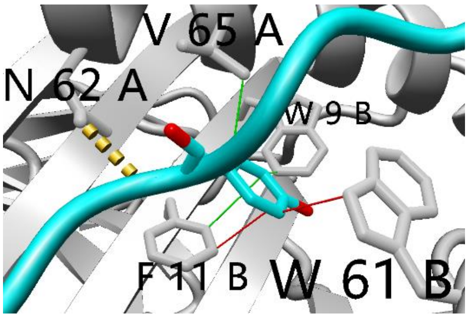

P6 negative: Y non-glia DQ2.5

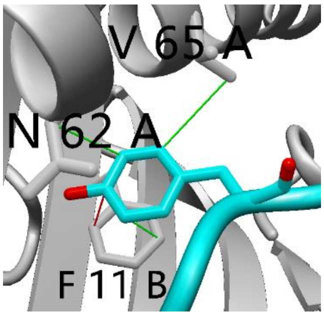

P6 negative: Y α-glia DQ8.1

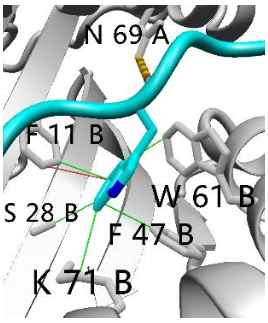

P7 positive: W α-glia DQ2.5

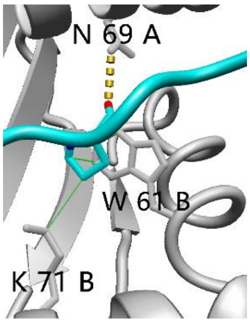

P7 positive: P non-glia DQ2.5

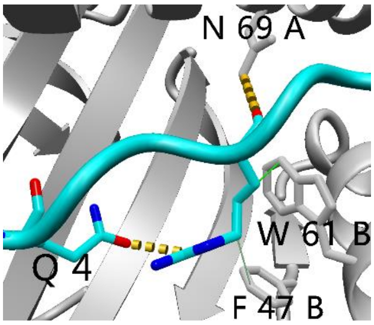

P7 negative: R α-glia DQ2.5

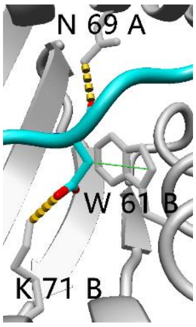

P7 negative: D non-glia DQ2.5

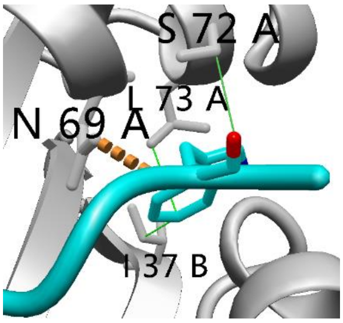

P9 positive: W α-glia DQ2.5

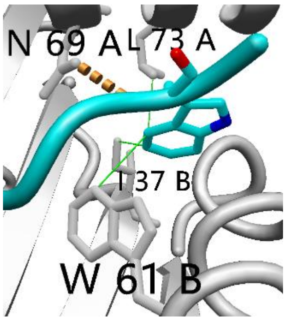

P9 positive: W non-glia DQ2.5

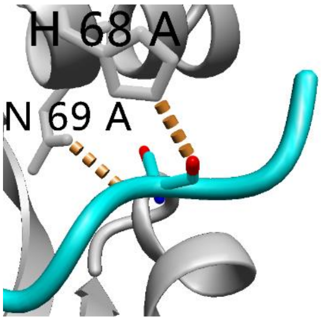

P9 positive: N α-glia DQ8.1

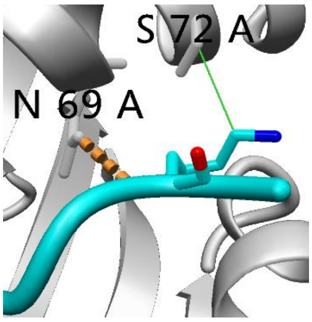

P9 negative: K α-glia DQ2.5

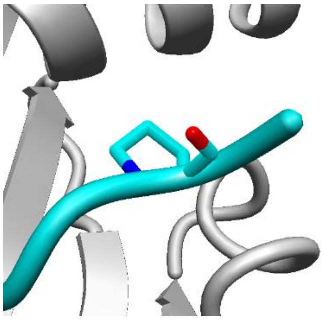

P9 negative: P non-glia DQ2.5

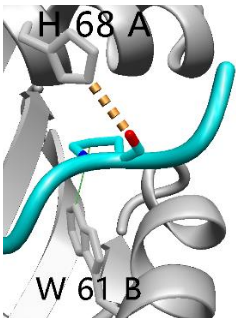

P9 negative: P α-glia DQ8.1

Figure S2. Intermolecular interactions between the most positively/negatively contributing peptide residues from α-gliadin and non-gliadin peptides binding into the corresponding pockets of HLA-DQ2.5 and HLA-DQ8.1. The hydrogen bonds are presented as orange discontinued lines; π-π – stacking – with red or pale pink for weaker contacts, hydrophobic interactions – with green or pale green for weaker interactions. The peptide backbone and side chains are colored in cyan. Hydrogens are not presented.
